# Evaluating an Incentive-Based mHealth App for Physical Activity Promotion Using the Obesity-Related Behavioral Intervention Trial Model: Small Cohort Study

**DOI:** 10.2196/85484

**Published:** 2026-04-10

**Authors:** Babac Salmani, Madison S Hiemstra, Harry Prapavessis, Leigh M Vanderloo, Marc S Mitchell

**Affiliations:** 1 School of Kinesiology, Faculty of Health Sciences, Western University, 1151 Richmond Street, London, ON, Canada, 1 519-661-2111; 2 ParticipACTION, Toronto, ON, Canada; 3 School of Occupational Therapy, Faculty of Health Sciences, Western University, London, ON, Canada

**Keywords:** mHealth, digital health, financial incentives, health behavior change, physical activity, behavioral economics

## Abstract

**Background:**

Physical inactivity remains a public health concern, with 42% (around 1 in 2) of women and 34% (around 1 in 3) of men in the United Kingdom, for example, failing to meet moderate-to-vigorous physical activity guidelines. To promote physical activity (PA) at scale, smartphone-based mHealth (mobile health) software apps offer a promising solution.

**Objective:**

This study aims to evaluate the feasibility of implementing an mHealth app offering very small (“micro”) financial incentives for PA in Leeds, United Kingdom.

**Methods:**

A 5-week single-arm proof-of-concept study was conducted with rolling recruitment among Caterpillar Health app users between September 12 and December 12, 2022 (Obesity-Related Behavioral Intervention Trial model, phase IIa). Users earned microincentives in the form of “points,” redeemable for consumer rewards (eg, movie tickets and gym passes), for meeting personalized daily step goals (US $0.13 per goal achieved; set using data from a 5-day baseline) and completing educational quizzes (US $0.33 per quiz). Descriptive statistics assessed feasibility outcomes (ie, reach, recruitment, retention, engagement, and acceptability) and preliminary effectiveness. Paired-samples *t* tests (*P*<.05) examined changes in weekly mean daily step count (from baseline) and step goal achievement over 5 weeks.

**Results:**

Of 285 app downloads, 46 users consented to participate (recruitment rate: 16.1%). Participants (mean age: 39.9, SD 11.1 y; 71.1%, 33/46 woman) had a baseline step count of 5598 (SD 2664) steps/day. A total of 25 participants remained engaged (ie, completed at least 1 quiz) at study week 5 (retention rate: 54.3%). Acceptability was high, with 75% of respondents (12/16) indicating they would recommend the app. Weekly mean daily step count did not significantly increase from baseline (mean difference 317, SD 2273, *P*=.53). Weekly daily step goal achievement rate (%) decreased from study week 1 to 5 (−23.23, SD 22.85, *P*=.02).

**Conclusions:**

Despite lower-than-expected recruitment and no statistically significant PA increase, relatively high engagement and acceptability suggest future pilot testing (Obesity-Related Behavioral Intervention Trial model, phase IIb) of a refined intervention (eg, wider selection of loyalty reward partners) and modified study protocol (eg, simplified consent process) is warranted.

## Introduction

Physical inactivity remains a public health concern, with 42% (around 1 in 2) of women and 34% (around 1 in 3) of men in the United Kingdom, for example, failing to meet moderate-to-vigorous physical activity guidelines [1]. To promote physical activity (PA) at scale, smartphone-based mHealth (mobile health) software apps offer a promising solution [2]. As global smartphone ownership continues to rise (ie, 85% in 2024 vs 66% in 2018 worldwide [3], with 87% of UK adults owning a smartphone in 2023) [4], mHealth apps may be one way of promoting population-level PA [5,6]. Despite their potential, low mHealth app engagement (eg, frequency and depth of use; a common precondition for mHealth app effectiveness) leading to little or no effect is typical [7]. Grounding mHealth app intervention in behavior change theory (eg, behavioral economics) may boost engagement and effectiveness [7-9]. For example, a systematic review of 35 randomized controlled trials (RCTs) by Boonmanunt et al [8] found that interventions informed by theory outperformed those that were not.

Behavioral economics, a branch of economics complemented by insights from psychology, has shown promise in boosting mHealth app engagement [10]. One of the central tenets in behavioral economics is that of “decision bias.” “Decision bias” refers to the systematic ways in which human decisions deviate from what would be considered rational or logical [11]. For example, “present bias” refers to the tendency for individuals to place disproportionate emphasis on the present costs and benefits of behaviors in decision-making while at the same time discounting future ones [11]. In the context of PA, for example, the immediate costs experienced with a bout of exercise (eg, discomfort) often outweigh future benefits (eg, lower cancer risk) in daily decision-making [12]. Financial incentives (FI), monetary rewards contingent on health behavior or outcome goal achievement, may encourage individuals to engage in more PA today, for instance, given the added short-term benefit of rewards [12]. There is evidence that FI-for-PA interventions can be effective [13,14]. For example, in a review of RCTs examining PA incentives, Mitchell et al [14] found that FI, worth on average about US $1.50 per day, produced short-term (ie, <6 mo), long-term (ie, ≥6 mo), and follow-up (ie, after incentive withdrawal) effects. While promising, these FI magnitudes may be cost-prohibitive when implemented at a population level, or longer-term (ie, more than 6 months, a theoretical threshold of behavior maintenance) [15]. This may be particularly true within the constraints of limited public health budgets [16]. For example, offering £1.00 (ie, US $1.33/d) per day to just 1% of the UK adult population (ie, 683,500 people) would cost £249.5 million (US $334.8 million) annually (ie, about 4.82% of the 2025 £3.85 billion [US $5.17 billion] public health budget) [17].

One way of controlling FI costs while maintaining their effects may be to limit the size of rewards, all the while maintaining integrity to behavioral economics—that is, offering FI close in time to the desired behavior or outcome to best leverage “present bias.” In the 2020 Mitchell et al [14] review, several RCTs offering very small (“micro”) FI increased PA (eg, US $0.09-US $0.53/d) [18-20]. For example, Rohde et al [18] offered microincentives (ie, US $0.09‐US $0.15/d) conditional on participants’ weekly gym attendance (ie, FI per day). These small cash rewards, given to both regular and irregular gym visitors, led to increased attendance in both the short- (ie, <12 wk) and long-term (ie, ≥12 wk) compared to a control group receiving unconditional FI. Much of the evidence supporting microincentive impact is derived from RCTs with limited external validity, however. Few have examined their impact in real-world public health settings [21-23]. Knowing more about microincentives outside of controlled experimental settings may enhance their impact in the public sphere.

One example of microincentives for PA deployed in the real world is Carrot Rewards (2015‐2019) [24], a Canadian mHealth app that offered microincentives for daily step goal achievement (eg, consumer loyalty points worth about US $0.03/day [ie, equivalent to 1 loyalty reward point] for movies, groceries, gas, etc). On one hand, the app saw high uptake and engagement, attracting more than 1.3 million downloads and 500,000 monthly active users and produced short- (ie, <6 mo) and long-term (ie, ≥6 mo) PA improvements [24]. On the other hand, higher than anticipated engagement resulted in large-scale daily step goal reward payouts (ie, totaling more than US $3 million over 4 years). This great cost, combined with reliance on government funds to pay for the microincentives [25], undermined the app’s scalability or sustainability potential and ultimately led to its discontinuation in 2019 [24]. Given the strong uptake, high engagement, and long-term effects seen with Carrot [16,24], efforts to replicate their microincentive approach, with some modification (eg, wean users off daily step goal rewards after a year), in different contexts are warranted. Specifically, examining how digitally delivered microincentives for PA may perform in a completely different country (ie, United Kingdom) with different partners (eg, loyalty point programs, government agencies, and nongovernmental organizations) and different users (eg, who may not respond to points or incentives the same way) [26] may yield valuable insights.

The purpose of this small 5-week proof-of-concept study, therefore, is to evaluate the feasibility of offering microincentives for PA in the United Kingdom via a commercial mHealth app. The primary indicators for this study are feasibility-related outcomes (eg, uptake, engagement, and acceptability), rather than statistically significant changes in PA. This study represents an important first step in a multiphased project aiming to develop, refine, and evaluate a promising (ie, more scalable and sustainable) FI-for-PA intervention in the United Kingdom.

## Methods

### Study Setting and Design

This was a small 5-week single-arm proof-of-concept study with rolling recruitment between September 12 and December 12, 2022. This research was conducted in partnership with Caterpillar Health, an mHealth app designed to support PA engagement through personalized daily step goals, evidence-supported educational content, and microincentives in Leeds, United Kingdom. This preparatory study was guided by the Obesity-Related Behavioral Intervention Trial (ORBIT) model [27], a structured framework that supports the systematic development and evaluation of behavioral interventions. The ORBIT model outlines staged phases, from early feasibility (phase I-II) to large-scale effectiveness testing (phase III-IV). This study represents phase IIa (ie, proof-of-concept), which emphasizes feasibility assessment as well as identifying opportunities to refine the intervention and future study design. To ensure the intervention and study design incorporated principles of equity, diversity, inclusion, and accessibility, the World Health Organization’s health equity toolkit [28] was used. This included appropriately describing categories of disadvantage (eg, gender, household income, and ethnicity), avoiding overly technical health language in both the app interface and recruitment materials, using inclusive imagery within the app, ensuring compatibility across different mobile operating systems (eg, Android [Google LLC] and iOS [Apple Inc]), and tailoring the approach to Leeds, United Kingdom (ie, a community with a relatively high prevalence of overweight and obese adults [63.9%]) [29] to ensure sensitivity to local health inequities. In line with the World Health Organization toolkit’s emphasis on participatory design, feedback from app users within the community was sought during this study to identify potential barriers and ensure cultural relevance. In addition, subgroup analyses were conducted to examine the impact of the interventions among equity-deserving groups (eg, individuals with low income, chronic conditions, or low baseline PA) to assess whether the strategies were equitably effective across diverse populations.

To promote the app, a paid advertising campaign on social media platforms (ie, Facebook [Meta], Instagram [Instagram from Meta], and Twitter [subsequently rebranded X; X Corp]) began on August 1, 2022 (ie, outside of this study’s period) to raise awareness of the app leading up to its launch on September 12, 2022. To further support app launch, Caterpillar collaborated with Leeds City Council to have emails sent to City employees, as well as to local KPMG (Klynveld Peat Marwick Goerdeler) employees and Hussle Gym members living in Leeds, United Kingdom, on August 8, 2022. Participation required several sequential steps, including app download and account registration. Informed consent to participate in this study was sought on 2 separate occasions. First, to authorize the collection of participants’ sociodemographics and app engagement data (starting September 12, 2022) through an in-app consent form for study participation, and then again separately (starting September 30, 2022) to authorize the collection of device-assessed daily step count data over a 5-day baseline followed by a 5-week intervention period. This aligned with the app’s “steps” feature launch on September 30, 2022, and captured participants’ daily step counts in the approximately 6 weeks following the second consent. Sociodemographic and health characteristics were collected through a survey embedded in the app (Multimedia Appendix 1). This trial was registered at ClinicalTrials.gov (NCT05294692). The STROBE (Strengthening the Reporting of Observational Studies in Epidemiology) statement checklist (Checklist 1) [30] for cohort studies is provided.

### Ethical Considerations

This trial was approved by Western University Health Sciences’ Research Ethics Board in March 2024 (Project ID: 120615). All participants provided informed consent before participation. Participant privacy and confidentiality were maintained through the use of deidentified data, secure data storage systems, and restricted access to study records. Participants were not compensated for study participation; however, as part of the intervention, they were eligible to earn FI contingent on achieving daily step goals. Incentives were provided in British pounds (£0.10), equivalent to approximately US $0.13, based on a conversion rate of £1=US $1.34 at the time of this study.

### Intervention

**Figure 1.**
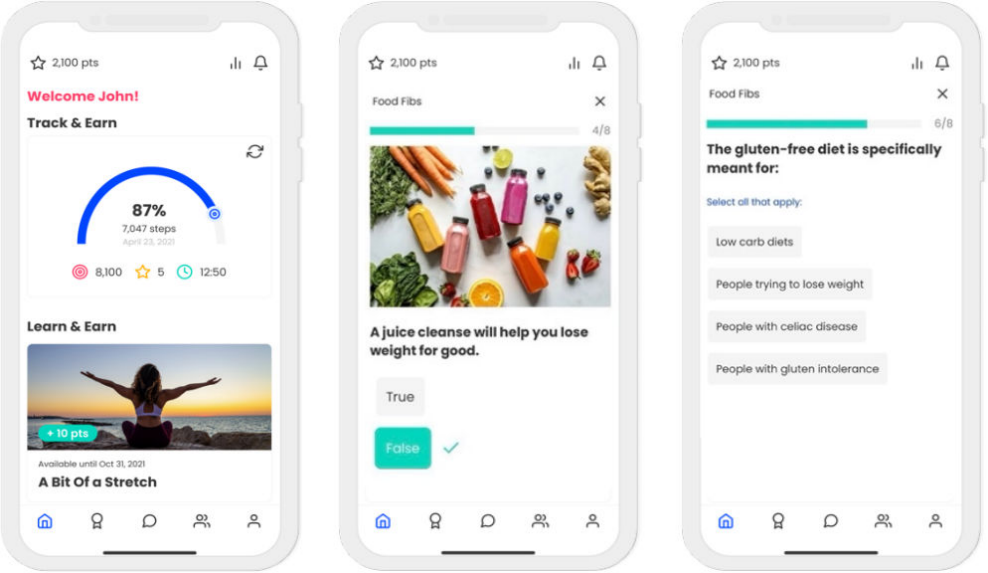
The Caterpillar Health mobile app allowed participants to monitor their PA, view progress toward goals, and interact with educational content designed to support engagement with the platform. PA: physical activity.

Caterpillar*’*s cornerstone feature, “personalized challenges,” offered microincentives for personalized daily step goal achievement (Figure 1). Users’ initial daily step goals were calculated as the median step count from the 5-day (ie, no goal and no microincentive) baseline period, where users were instructed to “wear their device” as much as possible. After this baseline, users could earn microincentives in the form of points (“incentives and rewards”; equivalent to US $0.13/d) for meeting their daily goals (ie, calculated as the median step count from the previous 7-day week). These points could be redeemed for consumer goods (ie, movie tickets from Vue Cinemas and gym passes from Hussle Gym). Users could also opt into weekly “step challenges” (ie, complete 5 out of 7 daily step goals) for additional points (US $0.33). Finally, users could also complete 4 educational quizzes per week (“health education and microlearning”; US $0.33/quiz). The educational quizzes primarily covered UK healthy eating and PA guideline content (in line with the National Health Service, “live well” guidelines). The app’s key features, along with their alignment to relevant behavioral economic constructs, are described in full in Multimedia Appendix 2.

### Outcomes

Several feasibility outcomes are reported, including (1) reach (ie, number of emails sent during app promotion), (2) recruitment (ie, number of app downloads and number of users providing initial informed consent) and retention (ie, proportion of participants completing an educational quiz in study week 5), (3) app engagement (ie, proportion of total possible educational quizzes completed during this study’s period), and (4) intervention acceptability (ie, exit survey responses informed by the validated Mobile Application Rating Scale [31,32]; Multimedia Appendix 1) data were collected. To examine the potential impact of the app on PA, device-assessed (5) baseline and weekly mean daily step count (ie, 1000 to 40,000 steps/day considered within an acceptable range) was collected over a roughly 6-week period (ie, 5 days plus 5 weeks following “steps” feature launch), while (6) daily step goal achievement (ie, proportion of days [out of 7] a user met or exceeded their daily step goals each week) was assessed over the 5-week intervention period.

### Theoretical Underpinning

The behavior change wheel (BCW) [33], an amalgamation of 19 behavior change theories, provides a structured and theory-driven framework for designing and evaluating behavioral interventions aimed at both short- and long-term behavior change. The BCW consists of 3 layers. At its core, the BCW defines behavior as an interaction between one’s capability (eg, knowledge and skills), opportunity (eg, access to facilities and social support), and motivation (eg, goal setting and rewards) to engage in a particular health behavior. This is referred to as the capability, opportunity, motivation–behavior (COM-B) model. The integration of the COM-B model with microincentives may help tackle barriers to PA (eg, lack of knowledge and low engagement) and enhance the effectiveness of future interventions to sustain behavior over time. To date, several commercially available PA apps (ie, Sweatcoin, Carrot Rewards, StepBet, etc) have incorporated behavior change theories (ie, self-determination theory) within their app design, alongside FI mechanisms (ie, gamified points and lotteries) to boost PA and drive user engagement. However, further research may be needed to test the effectiveness of microincentives in real-world settings. During the design phase of the Caterpillar Health app, the research team used the COM-B model as the theoretical foundation of the app to identify and address barriers and enablers to increase daily step counts and app engagement. Specifically, the model guided the identification of key influencing factors (ie, lack of motivation and time constraints) and facilitators (ie, social support and user-friendly interfaces). These insights were then used to inform how the app’s features could be designed to best elicit behavior change. The categories evaluate the intervention derived from the behavior change technique taxonomy, which systematically classifies 93 distinct techniques into 16 clusters used to design and evaluate behavior change interventions [34,35]. The app’s key features are described according to the COM-B model in Multimedia Appendix 2.

### Statistical Analysis

**Figure 2.**
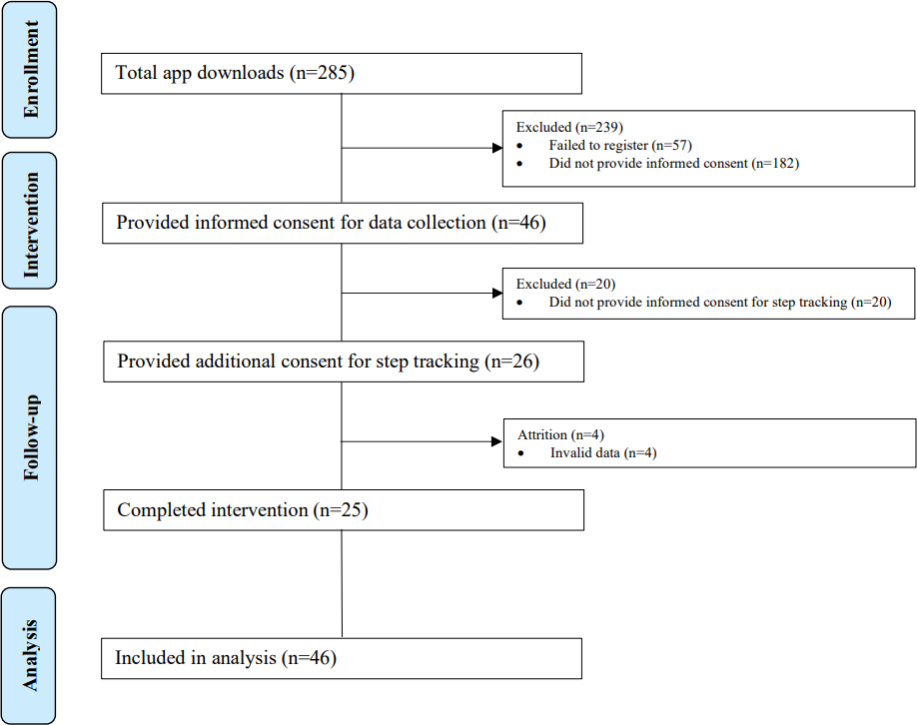
Flow chart of participants in a 6-week study with rolling recruitment between September 12 and December 12, 2022, using the Caterpillar Health app. Failed to register refers to users who attempted to sign up for the app but did not complete the registration process. Did not provide informed consent includes users who accessed the platform but did not complete the consent process required for participation in this study. Invalid data includes users with less than 4 days in a week with step counts in the acceptable range (ie, 1000 to 40,000) during the 5-week physical activity assessment period (step count data were included for the 22 users who provided consent for step tracking). Completed intervention represents users who completed at least 1 quiz in study week 5.

Statistical analysis was performed using SPSS (version 28.0.1.0; IBM Corp). Descriptive statistics were used to assess reach, recruitment, and retention rates, app engagement, and intervention acceptability. To assess preliminary effectiveness, paired-samples *t* tests were used to examine change in weekly mean daily step count from the 5-day baseline (vs study week 5) among the subsample of users downloading the app on or after September 30, 2022 (with “steps” feature included). Exploratory analyses also examined change in weekly mean daily step count by PA level (ie, physically active: ≥5000 steps/d at baseline; physically inactive: <5000 steps/d at baseline) [36], engagement (ie, high engagement: ≥50% of total quizzes completed [≥12 of 24 quizzes]; low engagement: <50% of quizzes completed [<12 of 24 quizzes]), and chronic condition status (ie, at least 1 chronic condition vs no chronic conditions). Given the small sample size in each of these subgroups Figure 2, the mean difference (MD) in weekly mean daily step counts and daily step goal achievement was reported descriptively. As this was a proof-of-concept study, a priori sample size calculation to detect statistical significance was not conducted. The results, however, will be used to inform sample size calculations for future pilot work. Nonetheless, statistical significance was measured as *P*<.05, and Cohen *d* for local effect sizes of weekly mean daily step counts were calculated, where *d*=0.2 indicates a small effect, *d*=0.5 a medium effect, and *d*=0.8 a large effect.

## Results

### Sample Characteristics

A total of 285 users downloaded the app, and 228 completed app registration. Of these, 46 users provided initial informed consent (Table 1). A subgroup of users also provided consent authorizing the collection of device-assessed daily step count data (n=22). Among the total sample, participants were primarily woman (71.1%, 32/45) with an average age of 39.9 years and a baseline daily step count of 5598 steps/day. Additionally, 65.7% (23/35) self-reported one or more chronic condition diagnoses, and 25% (9/36) reported at least one disability or impairment. Notably, a greater proportion of participants reported the use of a fitness tracker (65.9%, 27/41) and lower household income (18.9%, 7/37) compared to the general UK population (35% and 13%, respectively). These demographic characteristics may have influenced engagement and responsiveness to app-based microincentives, potentially limiting the generalizability of findings to broader populations (eg, more balanced gender distribution, lower fitness tracker use, and different health profiles).

**Table 1.** Baseline characteristics of the total sample, subgroup, and general UK population. Chronic condition defined as a diagnosis by a health professional with symptoms expected to last, or have already lasted 6 months or more, “total” sample includes participants who provided initial informed consent for data collection, “subgroup” sample includes participants who provided a separate informed consent for step count tracking. Household income of US $27,156/year is equivalent to the UK’s cutoff for low household income (£21,000/year).

Characteristics	Total (N=46)	Subgroup (n=22)	UK population [36,37] (n=67,749,091)
Age (years)	39.9 (11.1)^a^	43.2 (10.5)^a^	40.7^b^
Gender (woman)^c^, % (n/N)	71.1 (32/45)	86.4 (19/22)	51.0
Ethnicity (% White)^d^	80.5 (33/41)	95.5 (21/22)	81.7
BMI (kg/m^2^)	27.8	29.5	27.4
Chronic condition (one or more), % (n/N)	65.7 (23/35)	65.0 (13/20)	38.4
Diagnosed disability, % (n/N)	25.0 (9/36)	19.1 (4/21)	24.0
Housing location (urban), % (n/N)	89.2 (33/37)	95.5 (21/22)	84.2
Operating system (iOS), % (n/N)	62.2 (23/37)	95.5 (21/22)	50.8
Fitness tracker (yes), % (n/N)	65.9 (27/41)	77.3 (17/22)	35.0
Household income (below US $27,156/year), % (n/N)	18.9 (7/37)	5.3 (1/19)	13.0
Steps per day (baseline mean, SD)	5598 (2664)	5547 (2619)	5444 (N/A^e^)

a Mean (SD).

b As the UK population data were obtained from the Office for National Statistics (2021 Census), only the median age in years is available. The IQR is not reported in this source and therefore cannot be included.

c Gender was self-reported by participants based on their gender identity (e.g., man, woman).

d Participants selected ‘Caucasian’ in the original questionnaire; this has been reported as ‘White’ in the manuscript to reflect current terminology.

e N/A: not applicable.

### Main Findings

In total, over 15,000 and 3000 emails were sent to Leeds City Council and KPMG employees, respectively, while more than 7000 emails targeted Hussle Gym members. The number of social media impressions was not recorded. During this study’s period, Caterpillar recorded 285 app downloads, of which 46 (16%) provided informed consent and enrolled in this study. Retention at the end of the 5-week intervention was 54.3% (25/46 enrolled participants; Figure 2). Among participants providing initial consent, 95.6% (n=44) completed at least one educational quiz, and 39.1% (n=18) met the criterion for high engagement, defined as completion of ≥50% of available quizzes (≥12 of 24 quizzes). On average, participants completed 8.1 quizzes over the 5-week intervention period (out of 24 quizzes offered). Intervention acceptability was high, with most participants completing the exit survey reporting “good” to “excellent” app aesthetics (15/16, 93.7%), having learned new health information (14/16, 87.5%), and, notably, they would recommend the app to others (12/16, 75%). Constructive feedback focused on the lack of customizable features (eg, user input and sharing options; 2/16, 12.5%) and dissatisfaction with daily step goal presentation (eg, not visually engaging or personalized to the user’s preferences; 2/15, 13.3%).

**Figure 3.**
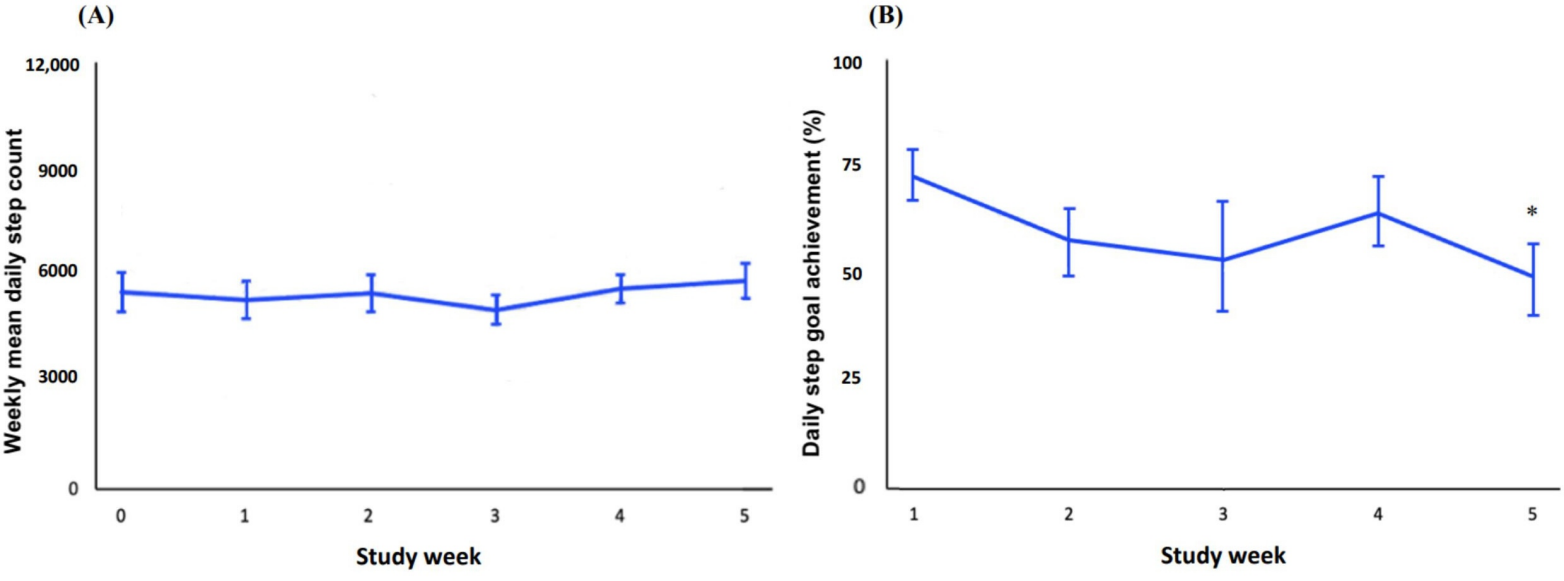
Weekly mean (A) daily step count and (B) daily step goal achievement with standard errors.

Weekly mean daily step count did not significantly increase from baseline (MD 317, SD 2273, *P*=.53; Figure 3). Moreover, weekly daily step goal achievement rate dropped over the 5-week period (MD −23.23, SD 22.85, *P*=.02). Among inactive participants (ie, <5000 steps at baseline), the weekly mean daily step count at baseline and study week 5 was 3509 (SD 1024) and 4656 (SD 2264), respectively (Multimedia Appendix 3). In contrast, active participants accumulated 7585 (SD 2053) steps/day at baseline and 7073 (SD 1753) at study week 5. Participants with at least one chronic condition had a mean daily step count of 5104 (SD 2533) at baseline and 5734 (SD 2434) at study week 5, whereas those without chronic conditions accumulated 6279 (SD 2932) and 5853 (SD 2379), respectively. Weekly step goal achievement rates were 74.2 (SD 18.6) at week 1 and 62.8 (SD 23.9) at study week 5 among inactive participants, and 76.2 (SD 16.5) and 33.3 (SD 8.3), respectively, among active participants. Those with chronic conditions reported 74.3 (SD 18.6) at week 1 and 54.2 (SD 27.5) at study week 5; those without chronic conditions reported 76.2 (SD 16.5) and 47.6 (SD 21.8), respectively.

## Discussion

### Principal Results

This single-arm, proof-of-concept ORBIT model phase IIa study assessed the feasibility of implementing Caterpillar, a commercial mHealth app offering microincentives for PA in Leeds, United Kingdom. Over 25,000 emails were sent on August 8, 2022, to raise awareness of the app launch on September 12, 2022. The app was downloaded 285 times during this study’s period, with approximately 15% (46/285) of users providing initial consent to participate in this study. Over the 5-week intervention, about 40% (18/46) of users were classified as very engaged (ie, completed ≥50% of quizzes [≥12 of 24 quizzes]). Intervention acceptability was high, with 75% (12/16) indicating they would recommend the app to others. Although Caterpillar did not appear to significantly increase PA, daily step goal achievement was fairly high in study week 1 (about 75%), along with exploratory subgroup trends (eg, for inactive adults), suggesting potential for impact. This finding should be interpreted in the context of a sample with relatively high baseline activity levels, which may have limited the scope for short-term, measurable increases in PA within this low-intensity, microincentive intervention. The discrepancy between high engagement and limited PA change may reflect several factors, including the short 5-week intervention duration, constraints in the incentive structure (eg, limited reward options), and potential limitations in how PA was measured (eg, reliance on daily step counts only). As this is a feasibility study, the emphasis is on effect sizes and feasibility outcomes (eg, engagement and acceptability) rather than on statistical significance; *P* values are reported for reference only. Taken together, these findings suggest the delivery of PA microincentives via Caterpillar in Leeds is feasible, with PA promotion potential given relatively high engagement and favorable subgroup trends. Pilot testing to assess the preliminary effectiveness of a refined intervention and study protocol is warranted (ie, phase IIb).

Several opportunities to improve the intervention and study protocol were identified (Table 2). For example, under “rewards and threats” in Table 2, only 2 corporations (ie, Vue Cinemas and Hussle Gym) partnered with Caterpillar to deliver consumer rewards that could be redeemed through the app, potentially limiting the app’s appeal. Partnerships with a wider array of corporations to provide more rewards options may help boost uptake in the future, as suggested by studies demonstrating that perceived reward value may help drive mHealth app engagement (eg, Are rewards meaningful to the target audience?) [38,39]. Under “feedback and monitoring” in Table 2, the app targeted any-intensity daily step count to, in part, promote participant autonomy and the potential for sustained change. Future iterations could broaden the intervention to promote a wider range of PA intensities (eg, moderate-to-vigorous physical activity and high-intensity interval training) and behaviors (eg, cycling, resistance training, and sedentary behavior reduction). In addition, under “associations” in Table 2, participant feedback highlighted limited opportunities for customization (eg, setting routine-based reminders and personalized push notifications), which will directly inform future iterations of Caterpillar to enhance usability and sustained engagement. From a study protocol perspective, several enhancements could strengthen future iterations of this intervention. First, more active recruitment strategies (eg, in-person presentations and text message invitations) could be used in place of the more passive approaches used here (eg, company-wide emails and social media campaigns) [40,41]. Second, the current goal-setting approach, which relies on the user’s median daily step count from a 5-day baseline and a 7-day window for each subsequent week, may be suboptimal, particularly given the observed decline in step goal achievement over time. Short baselines may not fully capture typical variability in daily step patterns, potentially resulting in goals that are too easy or too difficult and limiting motivation [33]. Future trials could improve goals by using a longer baseline period (eg, 7 days), rolling averages across multiple weeks (eg, median step count over the past 2 or 3 weeks), and goals that account for variability in step count (eg, account for both median step count and SDs), potentially offering better daily goals [42,43]. Finally, the current single-arm design limits the ability to draw causal inferences regarding the intervention’s effectiveness. Later phases of research could include a pilot RCT (ie, ORBIT phase IIb) to allow for preliminary testing of intervention effectiveness and to inform the design of future fully powered trials [27].

**Table 2.** Intervention and study protocol areas for improvement and recommendations. Valid data includes users with daily step counts in an acceptable range (ie. 1000 to 40,000). The categories evaluating the intervention derive from the behavior change technique taxonomy [34,35].

	Areas for improvement	Evidence-informed recommendations
Intervention		
Associations	Few prompts or contextual cues to reinforce behavior	Introduce customizable push notifications, routine-based reminders, prompts (eg, after meals), and environmental cues (eg, “take the stairs instead of the elevator”) [44]
Comparison of behavior	No social comparison or benchmarking	Add anonymized leaderboards, user averages, or percentile rankings to show how user performance compares with peers [45]
Feedback and monitoring	Only step count tracked; PA^a^ intensity, duration, and type were not captured	Expand intervention to include other PA types (eg, cycling, resistance training, and sedentary behavior reduction) [46] Display personalized insights (eg, comparative analysis of PA behavior to users’ previous week) [47]
Repetition and substitution	Limited support for habit formation or alternative PA routines	Include streak tracking and options to substitute missed step goals with alternative PA tasks (eg, cycling) [48]
Rewards and threats	Limited consumer reward options (n=2) Boost microincentive impact with promising incentive designs	Offer additional reward options (eg, grocery, transit, and entertainment) [49] Test lost-framed incentives [49]
Shaping knowledge	Educational content delivered only through static quizzes and educational content	Incorporate interactive learning modules, infographics, video explainers, and progressive unlocking of content based on user performance [50]
Social support	No mechanisms to connect users or enable peer interaction	Add features such as group challenges, friend invitations, discussion boards, or accountability buddies [51]
Study protocol		
Study setting	Limited generalizability beyond the Leeds, United Kingdom context	Replicate procedures in diverse geographies (eg, urban and rural North American cities) [47]
Study design	Single-arm design limits causal inference (ie, no comparator group to isolate app effects)	Consider (pilot) RCT^b^ design, or incorporate a waitlist condition, in future phases for improved causal inference [27]
Recruitment	Reliance on passive outreach strategies (eg, social media and company-wide email) Lack of tailored messaging across channels Rolling recruitment may lead to variability in exposure time and seasonal bias Staggered consent process may have confused participants, limited recruitment rate	Add more active recruitment, such as at community health events or workplace briefings, to reach underserved groups (eg, low-income adults) [52] Customize messaging based on sociodemographics (eg, younger adults via Instagram and older adults via email with testimonials) [52] Standardize recruitment windows or stratify analysis by recruitment timing to reduce confounding [53] Combine consents or replace with an integrated consent process [54]
Sample	Limited sample diversity (eg, 71% [32/45]woman, 81% [33/41] White ^c^) High prevalence of fitness tracker use (65.9% [27/41]) may limit broad applicability Small sample size (n=46; n=22)	Consider oversampling underrepresented groups or tailoring eligibility to target populations (eg, low-income or visible minority groups) using stratified sampling (ie, randomly sample participants from distinct strata [eg, by age group and gender]) [55] Prioritize strategies to engage individuals who do not have access to extra digital tools at baseline (eg, fitness trackers) [55]
Outcome assessment	Calculation of users’ initial daily step goals (median step count from the 5-day baseline) may not be ideal No data collected beyond 6 weeks of app use Sole reliance on quiz completion for engagement measurement	Use a full 7-day baseline period using only valid data to better represent habitual PA patterns and account for weekday or weekend differences [56] Add postintervention follow-up to assess maintenance or motivation [57] Track additional engagement metrics (eg, number of app opens, time spent per open, and reward redemption rates) [58]
Analysis	Paired sample *t* test limits the ability to model time trends and handle missing data No mediation or moderation analyses conducted	Use linear mixed model in the future to model repeated measures [59] Explore mediation pathways (eg, goal → engagement → PA) [60]

a PA: physical activity.

b RCT: randomized controlled trial.

c Participants selected ‘Caucasian’ in the original questionnaire; this has been reported as ‘White’ in the manuscript to reflect current terminology.

Despite fairly high engagement and acceptability, the lack of a significant increase in weekly mean daily step count may reflect a combination of declining adherence over time, relatively high baseline activity levels, and aspects of the intervention design that limited short-term gains in PA. The sample’s mean baseline step count (5598, SD 2664 steps/day) was slightly higher than the UK population average (5444 steps/day), suggesting participants may have been more physically active at enrollment than the general population. This raises the possibility of a ceiling effect, whereby participants had less capacity for observable improvement over a short intervention period, which may partly explain the absence of a statistically significant increase in PA. Several observations warrant further consideration. First, no immediate increase in PA was observed from baseline to study week 1, contrary to findings from earlier FI interventions where behavioral effects influence PA behavior immediately and tend to decrease gradually [24,25]. This observed delay may reflect the time required for users to build awareness, navigate app features, and begin integrating new routines [24]. In a future ORBIT phase IIb study, this initial delay could be addressed by incorporating a structured onboarding process that includes guided app walkthroughs, early incentives for app exploration, and prompts to encourage immediate goal engagement. These strategies may help accelerate user activation, reduce the learning curve, and facilitate earlier behavior change. Second, the apparent increase in PA among low-active users and decrease among high-active users should be interpreted cautiously. These subgroup trends are hypothesis-generating and may primarily reflect regression to the mean (ie, a statistical tendency for extreme values to move closer to the average over time) rather than a true intervention effect, particularly in the absence of inferential statistical testing [61,62]. A future ORBIT phase IIb study may protect against this alternative explanation by including a comparison group to better distinguish intervention effects from natural variability. Third, weekly step goal achievement was initially high but declined over the 5-week intervention. Declining goal achievement may have been due to fatigue (ie, physical capability), poorer weather (ie, opportunity), or decreasing perceived value of incentives over time (ie, motivation), and importantly, the mechanics of goal recalibration, which may have unintentionally made goals more difficult over time. A future ORBIT phase IIb trial may address these factors by incorporating adaptive intervention strategies (eg, offering users the option to modify goals during periods of low capability [eg, illness and fatigue]), integrating weather-responsive prompts or indoor activity suggestions to mitigate environmental barriers, and applying variable incentive structures (eg, loss-framed) to sustain motivation over time. These refinements would help test the intervention under more dynamic, real-world conditions and better assess its potential for long-term adherence.

### Comparison With Prior Work

Findings should also be considered in light of similar literature. The current study’s recruitment rate (≈15% [46/285]) was lower than rates reported in similar feasibility trials of digital health interventions promoting PA with microincentives, which have ranged from 21% to 78% [23,26,63]. Several factors may have contributed to this suboptimal conversion to study participation, including passive recruitment strategies (eg, social media campaigns), limited diversity in reward offerings, and participant burden associated with a multistep consent process. Nonetheless, the observed retention rate (about half) and level of engagement (40% [18/46] of users completing ≥50% of available quizzes [≥12 of 24 quizzes]) were higher than rates typically reported with prior digital health interventions, where engagement, more broadly defined as state-like construct that refers to the extent (eg, amount, frequency, duration, and depth) of usage, typically ranges between 20% and 30% [58]. Notably, higher app engagement has been linked to greater intervention effects [8,38], suggesting that the fairly high engagement rate observed here (40%) is encouraging from a health behavior change perspective. Favorable exit survey responses align with previous evidence suggesting that personalized designs (eg, personalized daily step goals) and small, immediate microincentives enhance intervention acceptability [24,64]. Importantly, the sample included a relatively high proportion of low-income participants (18.9%) compared to the national average in the United Kingdom (13%). This is notable given that low-income populations often encounter more barriers to PA engagement compared to high-income groups [65]. Although significant PA changes were not observed in this feasibility study, exploratory subgroup descriptive summaries suggest potential among higher-risk groups (eg, inactive participants and those living with chronic conditions). These possible trends are consistent with prior research indicating that microincentives may be particularly effective in promoting PA among at-risk populations [13,18].

### Limitations

This study has several strengths. Step data was collected using validated step tracking devices (ie, smartphones and fitness trackers in free-living conditions) [66], and the app’s cost-free design may have appealed more to lower-income, higher-risk users. Nonetheless, several limitations warrant consideration. First, this study’s low recruitment rate limited the sample size and statistical power to detect meaningful PA changes, as well as sample diversity. The requirement for 2 separate consents (ie, one through the app’s onboarding system [n=46] and another within the app for step count data collection [n=22]) likely reduced participant recruitment. This 2-stage structure reflected a separation between consent for data collection of standard app use and consent for step count–specific data collection, a design that was partly inherent to the app’s real-world deployment. However, future trials may benefit from integrating or streamlining consent procedures to minimize participant burden and improve enrollment efficiency in digital health interventions. One practical approach is the adoption of a tiered, integrated digital consent model, in which participants provide initial consent for minimal, low-risk data collection (eg, nonidentifiable engagement metrics) during onboarding, followed by feature-specific (eg, linking a wearable device), just-in-time consent prompts delivered contextually. This sandbox-style consent approach maintains ethical transparency and participant autonomy while reducing upfront friction and represents an appropriate, measurable methodological refinement suitable for evaluation in a subsequent ORBIT phase IIb study. Second, rolling participant recruitment meant the approximately 6-week evaluation window varied somewhat by participant (eg, some participant data collected in temperate September-October and some in cooler November-December), potentially introducing seasonal (eg, weather) and history bias [64]. Third, if participants did not carry their smartphones or wearable devices consistently throughout the days and weeks, there may be gaps in the data recorded at baseline and during the intervention period [66]. These gaps may lead to an underestimation of the actual number of steps taken per day (ie, measurement bias). However, incentivization may have addressed this by increasing the likelihood that participants consistently carry their devices during the intervention. Fourth, this feasibility study used a single-arm design without a control group, limiting causal inference. Without a comparator, it is difficult to determine whether observed outcomes are attributable to the intervention itself or to external factors (eg, seasonal changes and concurrent health campaigns). Fifth, although the current study’s approach to assessing engagement aligns with recommended practices for reporting mHealth usage data (eg, macro-level of engagement) [67] and user-reported acceptability (eg, exit survey responses) [31], the proxy used may not fully capture the complexity of user engagement [68]. Alternative metrics (eg, frequency, duration, and other feature use) could have provided a more nuanced understanding. Such depth is important for identifying which components were most engaging, informing iterative improvements, enhancing user retention, and elucidating potential mechanisms linking engagement to PA outcomes. Without these insights, key patterns (eg, points of disengagement) may remain undetected [68]. Sixth, although the target behavior chosen for this app was to significantly increase users’ daily steps, it is important to note that there are other behaviors related to movement that could be targeted. For example, in addition to encouraging users to walk or run more, the app could also support users to be less sedentary (ie, sit less) or to increase other moderate-to-vigorous intensity activities (eg, cycling to work). Seventh, although step goals were recalculated weekly based on prior step data (eg, median step count from the previous 7-day week), no explicit minimum or maximum thresholds were applied; goals were determined entirely by participant data, without the use of predefined minimum or maximum thresholds or manual adjustments. This may have contributed to potential plateau effects in participant motivation and engagement over time [69]. Eighth, participants exhibited relatively high baseline engagement with PA and self-monitoring behaviors. Mean baseline daily step counts exceeded national averages, and 65.9% of participants reported using a fitness tracker at baseline (ie, nearly double the UK population prevalence [≈35%]). This suggests that the sample may have been more motivated, health-conscious, or technologically engaged than the general population. Such preexisting engagement may have positively influenced app uptake, sustained use, and favorable acceptability ratings, and therefore, the high engagement and acceptability observed may not fully generalize to populations with lower baseline activity levels or less familiarity or interest in digital health technologies. Finally, in its current form, Caterpillar is not suitable for people who cannot adequately read and comprehend English, limiting its generalizability, particularly in diverse urban settings such as Leeds, where nearly 10% of adults report a first language other than English [70].

### Conclusions

This ORBIT model phase IIa study served to assess the feasibility of the intervention and refine the delivery and examination of the Caterpillar app that offered microincentives to boost PA in the United Kingdom. Relatively high app engagement and acceptability, and preliminary evidence of PA increases, support advancing to a more robust (ie, 2-arm) pilot evaluation.

## Supplementary material

10.2196/85484 Multimedia Appendix 1 Demographic and health survey, exit survey questions, and responses.

10.2196/85484 Multimedia Appendix 2 Caterpillar health BCTs (behavior change technique) and COM-B (capability, opportunity, motivation-behavior).

10.2196/85484 Multimedia Appendix 3 Baseline characteristics of subgroup samples.

10.2196/85484 Checklist 1 STROBE statement checklist.

## Abbreviations:

BCW: behavior change wheel
COM-B: capability, opportunity, motivation–behavior
FI: financial incentive
KPMG: Klynveld Peat Marwick Goerdeler
MD: mean difference
mHealth: mobile health
ORBIT: Obesity-Related Behavioral Intervention Trial
PA: physical activity
RCT: randomized controlled trial
STROBE: Strengthening the Reporting of Observational Studies in Epidemiology
