# Supplementary material for: Evaluating an Incentive-Based mHealth App for Physical Activity Promotion Using the Obesity-Related Behavioral Intervention Trial Model: Small Cohort Study
Source: JMIR Form Res. 2026 Apr 10;10:e85484. doi: 10.2196/85484 (PMC13068306; doi:10.2196/85484)
Supplement: Multimedia Appendix 1 — Demographic and health survey, exit survey questions, and responses. [file formative-v10-e85484-s001.docx]

**Table S1.** Sociodemographic and health survey questions and response options.

| **Question** | **Responses** |
| --- | --- |
| 1. How old are you? | [Text entry field] |
| 2. What is your gender? | a. Nonbinary  b. Female  c. Male  d. Other  e. Prefer not to answer |
| 3. How much do you weigh (kg)? | [Text entry field] |
| 4. How tall are you (meters)? | [Text entry field] |
| 5. Have you ever been diagnosed with any disability or impairment? Which of the following have been diagnosed? | a. A sensory impairment (vision, hearing)  b. A mobility impairment  c. A learning disability (ADHD, dyslexia)  d. A mental health disorder  e. A disability or impairment not listed above  f. Prefer not to answer  g. No, I have not been diagnosed with a disability or impairment  h. Prefer not to answer |
| 6. Do you have any of the following chronic illnesses or conditions? These are conditions diagnosed by a health professional that are expected to last, or have already lasted 6 months or more. | a. Arthritis (rheumatoid and osteoarthritis)  b. Osteoporosis  c. Asthma  d. COPD  e. Angina f. Heart attack  g. Neurological disease  h. Diabetes Type I or II  i. Depression  j. Anxiety/panic disorders  k. Hearing impairment  l. Obesity, BMI >30kg/m2  m. Chronic illness not listed  n. No, I have not been diagnosed with a chronic disease  o. Prefer not to answer |
| 7. Including yourself, how people live in your household? | a. More than 5 people  b. 5 people  c. 4 people  d. 3 people  e. 2 people  f. 1 person  g. Prefer not to answer |
| 8. What type of accommodation do you currently live in? | a. Rented from Housing Association  b. Owned with a mortgage or loan  c. Owned outright (without a mortgage)  d. With parents or relatives  e. Rent free  f. Rented from someone else  g. Other  h. Prefer not to answer |
| 9. Is the house or flat in which you live... | a. Urban (city or downtown)  b. Suburban (residential area outside a city)  c. Rural (countryside, small town, or farming area)  d. Prefer not to answer |
| 10. What is the highest level of education you have completed? | a. O Level or GCSE equivalent (Grade A-C) or O Grade/CSE equivalent (Grade 1) or Standard Grade Level 1-3  b. ONC/National Level BTEC  c. At school part-time, A-Levels or Higher  d. Degree level qualification (or equivalent)  e. A part-time course at university, or college, including day release and block release  f. No formal qualification  g. Prefer not to answer   \|  \| \| --- \| |
| 11. What is your total household income before tax in the past year? | a. Up to £5,199  b. £5,200 to £10,399  c. £10,400 to £15,599  d. £15,600 to £20,799  e. £20,800 to £25,999  f. £26,000 to £31,199  g. £31,200 to £36,399  h. £36,400 to £51,999  i. £52,000 and above  j. Prefer not to answer |
| 12. Do you regularly use a wearable fitness tracker (e.g., Fitbit, Apple Watch)? | a. Yes  b. No  c. Prefer not to answer |
| 13. What operating system do you currently use? | d. iOS (Apple)  e. Android (Google)  f. Other  g. Prefer not to answer |
| 14. Which of the following best describes your ethnic background? | a. Pakistani  b. African  c. Caribbean  d. Arab  e. Chinese  f. Indian  g. Bangladeshi  h. White and Black African  i. White and Black Caribbean  j. White - Irish  k. White and Asian  l. White - Gypsy or Irish Traveller  m. White - English / Welsh/ Scottish/ Northern Irish/ British  n. Any other Asian background  o. Any other Black/ African/ Caribbean background  p. Any other White background  q. Any other ethnic group (or mixed/multiple)  r. Prefer not to answer |
| 15. What is your current employment status? | a. Retired  b. Not in the paid workforce (homemaker, looking after family)  c. Permanently unable to work because of long-term sickness or disability  d. Intending to look for work but prevented by temporary sickness or injury (max 28 days)  e. Unemployed, but looking for work  f. Student  g. Employed part-time, that is, less than 35 hours per week  h. Employed full-time, that is, 37.5 more hours per week  i. Prefer not to answer |
| 16. What is your marital/relationship status? | a. Widowed  b. Civil partnership  c. Separated or equivalent  d. Single  e. Married or equivalent (i.e., common law)  f. Divorced  g. Prefer not to answer |

*Note.* National Statistics Census 2021 (Government UK, 2021).

**Table S2.** Exit survey questions and responses.

| **Question** | **Responses** |
| --- | --- |
| 1. Does app have specific, measurable and achievable goals (specified in app store description or within the app itself)? | 1. App has no chance of achieving its stated goals  2. Description lists some goals, but app has very little chance of achieving them  3. OK. App has clear goals, which may be achievable  4. App has clearly specified goals, which are measurable and achievable  5. App has specific and measurable goals, which are highly likely to be achieved |
| 2. Does it allow user input, provide feedback, contain prompts (reminders, sharing options, notifications, etc.)? | 1. No interactive features and/or no response to user interaction  2. Insufficient interactivity, or feedback, or user input options, limiting functions  3. Basic interactive features to function adequately  4. Offers a variety of interactive features/feedback/user input options  5. Very high level of responsiveness through interactive features/feedback/user input options |
| 3. How accurately/fast do the app features (functions) and components (buttons/menus) work? | 1. App is broken; no/insufficient/inaccurate response (e.g. crashes/bugs/broken features, etc.)  2. Some functions work, but lagging or contains major technical problems  3. App works overall. Some technical problems need fixing/Slow at times  4. Mostly functional with minor/negligible problems  5. Perfect/timely response; no technical bugs found |
| 4. How easy is it to learn how to use the app; how clear are the menu labels/icons and instructions? | 1. No/limited instructions; menu labels/icons are confusing; complicated  2. Useable after a lot of time/effort  3. Useable after some time/effort  4. Easy to learn how to use the app (or has clear instructions)  5. Able to use app immediately; intuitive; simple |
| 5. How good does the app look? | 1. No visual appeal, unpleasant to look at, poorly designed, clashing/mismatched colours  2. Little visual appeal, poorly designed, bad use of colour, visually boring  3. Some visual appeal, average, neither pleasant, nor unpleasant  4. High level of visual appeal, seamless graphics, consistent and professionally designed  5. As above + very attractive, memorable, stands out; use of colour enhances app features/ menus |
| 6. Is app content correct, well written, and relevant to the goal/topic of the app? | 1. Irrelevant/inappropriate/incoherent/incorrect  2. Poor. Barely relevant/appropriate/coherent/may be incorrect  3. Moderately relevant/appropriate/coherent/and appears correct  4. Relevant/appropriate/coherent/correct  5. Highly relevant, appropriate, coherent, and correct |
| 7. Is the app interesting to use? Does it use any strategies to increase engagement by presenting its content in an interesting way? | 1. Not interesting at all  2. Mostly uninteresting  3. OK, neither interesting nor uninteresting; would engage user for a brief time (<5 minutes)  4. Moderately interesting; would engage user for some time (5-10 minutes total)  5. Very interesting, would engage user in repeat use |
| 8. Would you recommend this app to people who might benefit from it? | 1. (Not at all) I would not recommend this app to anyone  2. There are very few people I would recommend this app to  3. (Maybe) There are several people whom I would recommend it to  4. There are many people I would recommend this app to  5. (Definitely) I would recommend this app to everyone |

*Note.* Mobile Application Rating Scale  (Stoyanov et al., 2015).
