# Supplementary material for: Evaluating an Incentive-Based mHealth App for Physical Activity Promotion Using the Obesity-Related Behavioral Intervention Trial Model: Small Cohort Study
Source: JMIR Form Res. 2026 Apr 10;10:e85484. doi: 10.2196/85484 (PMC13068306; doi:10.2196/85484)
Supplement: Multimedia Appendix 2 — Caterpillar health BCTs (behavior change technique) and COM-B (capability, opportunity, motivation-behavior). [file formative-v10-e85484-s002.docx]

**Intervention**

Prior to download, users were required to have an app compatible smartphone (i.e., iPhone 5S model or higher, or an Android version that supports step count tracking [i.e., include a ‘built-in’ accelerometer]) and have the Health Kit or Google Fit application (or equivalent) downloaded. To complete app registration, users were asked to enter their birth year, gender, and postcode.

**Table S1.** COM-B components, definitions, and *Caterpillar Health* examples with related behaviour change techniques.

| **COM-B Component** | **Definition** | **Example** | **BCT** |
| --- | --- | --- | --- |
| 1a. Capability (physical) | Refers to the user’s physical ability to engage in a behavior (e.g., health tracking, exercise). | The app synchronizes with wearables and smartphones to track physical activity, steps, and sleep, providing real-time feedback on physical progress. | 2.2, 2,3 |
| 1b. Capability (psychological) | Refers to the user’s mental ability (e.g., knowledge, self-regulation). | The app delivers bite-sized educational content on nutrition, stress management, and fitness to enhance users' understanding and decision-making. | 4.1, 5.1 |
| 2a. Opportunity (social) | External social factors that enable or hinder behavior change (e.g., social support, group dynamics). | The app offers a visual progress tracker that can be shared with other users as well as friends and family outside the app, helping foster motivation and accountability. | 3.1, 3.2 |
| 2b. Opportunity (physical) | Environmental factors that support or hinder behavior change (e.g., tools, devices, availability of resources). | The app integrates with wearables to track health metrics like steps, sleep, and stress, providing continuous support for health monitoring. | 7.1, 12.5 |
| 3a. Motivation (reflective) | Involves cognitive processes, such as thinking, planning, and evaluating (e.g., goal setting). | Personalized progress tracking helps users reflect on their goals and achievements, reinforcing motivation to continue improving. | 1.1, 1.5, 2.2 |
| 3b. Motivation (automatic) | Involves emotional, habitual, or instinctive responses to cues (e.g., rewards, incentives). | The app provides rewards (e.g., points for completing tasks), creating a positive feedback loop that encourages continued engagement and healthy behaviour. | 10.1, 10.4 |

*Note. BCT*, Behaviour Change Technique (Michie et al., 2013). *BCW*, Behaviour Change Wheel (Michie et al., 2013)
