# Supplementary material for: Evaluating an Incentive-Based mHealth App for Physical Activity Promotion Using the Obesity-Related Behavioral Intervention Trial Model: Small Cohort Study
Source: JMIR Form Res. 2026 Apr 10;10:e85484. doi: 10.2196/85484 (PMC13068306; doi:10.2196/85484)
Supplement: Multimedia Appendix 3 — Baseline characteristics of subgroup samples. [file formative-v10-e85484-s003.docx]

**Table S1.** Baseline characteristics of subgroup sample (active vs. inactive population).

|  | Active | Inactive |
| --- | --- | --- |
| Sample size | 11 | 11 |
| Age years (mean, SD) | 43.5 (10.8) | 44.0 (10.3) |
| Gender (% female) | 90.9 | 81.8 |
| Ethnicity (% Caucasian) | 100.0 | 90.9 |
| Body Mass Index (BMI) | 30.6 | 28.2 |
| Chronic condition (% one or more) | 54.5 | 80.0 |
| Diagnosed disability (%) | 100.0 | 36.4 |
| Housing location (% urban) | 81.8 | 77.8 |
| Type of smartphone (% iOS) | 9.9 | 100.0 |
| Fitness tracker (% yes) | 81.8 | 72.7 |
| Median household income (% below $27,156 USD/year) | 18.2 | 9.1 |
| Steps per day (baseline mean, SD) | 7585 (2054) | 3508 (1024) |

*Note*. *SD* standard deviation, Physically active participants were those accumulating ≥5,000 steps/day at baseline whereas physically inactive participants were those accumulating <5,000 steps/day at baseline, Household income of $27,156 USD/year is equivalent to the UK’s cut off for low household income (£21,000/year).

**Table S2.** Baseline characteristics of subgroup sample (chronic vs. non-chronic condition).

|  | Chronic | Non-chronic |
| --- | --- | --- |
| Sample size | 13 | 9 |
| Age years (mean, SD) | 43.7 (9.4) | 43.8 (11.9) |
| Gender (% female) | 84.6 | 88.9 |
| Ethnicity (% Caucasian) | 100.0 | 88.9 |
| Body Mass Index (BMI) | 28.1 | 31.7 |
| Diagnosed disability (%) | 23.1 | 11.1 |
| Housing location (% urban) | 75.0 | 87.5 |
| Type of smartphone (% iOS) | 92.3 | 100.0 |
| Fitness tracker (% yes) | 69.2 | 88.9 |
| Median household income (% below $27,156 USD/year) | 0.0 | 37.5 |
| Steps per day (baseline mean, SD) | 5111 (2529) | 6176 (2768) |

*Note*. *SD* standard deviation, Chronic condition defined as a diagnosis by a health professional with symptoms expected to last, or have already lasted 6 months or more, Household income of $27,156 USD/year is equivalent to the UK’s cut off for low household income (£21,000/year).

**Table S3.** Baseline characteristics of subgroup sample (high vs. low engagement).

|  | High engagement | Low engagement |
| --- | --- | --- |
| Sample size | 19 | 3 |
| Age years (mean, SD) | 45.1 (10.1) | 36.0 (9.5) |
| Gender (% female) | 89.5 | 66.6 |
| Ethnicity (% Caucasian) | 100.0 | 33.3 |
| Body Mass Index (BMI) | 29.7 | 28.3 |
| Chronic condition (% one or more) | 72.2 | 33.3 |
| Diagnosed disability (%) | 21.1 | 0.0 |
| Housing location (% urban) | 77.8 | 100.0 |
| Type of smartphone (% iOS) | 100.0 | 66.6 |
| Fitness tracker (% yes) | 73.7 | 100.0 |
| Median household income (% below $27,156 USD/year) | 17.6 | 0.0 |
| Steps per day (baseline mean, SD) | 5295 (2248) | 7138 (4707) |

*Note*. *SD* standard deviation, Participants were classified as highly engaged if they completed ≥50% of the total quizzes whereas those completing <50% were considered to have low engagement, Household income of $27,156 USD/year is equivalent to the UK’s cut off for low household income (£21,000/year).
