# Supplementary material for: Evaluating an Incentive-Based mHealth App for Physical Activity Promotion Using the Obesity-Related Behavioral Intervention Trial Model: Small Cohort Study
Source: JMIR Form Res. 2026 Apr 10;10:e85484. doi: 10.2196/85484 (PMC13068306; doi:10.2196/85484)
Supplement: Checklist 1 — STROBE statement checklist. [file formative-v10-e85484-s004.pdf]

Supplementary File B

S2. STROBE Statement—checklist of items that should be included in reports of observational studies

	Item No.	Recommendation	Page No.	Relevant text from manuscript
**Title and abstract**	1	(*a*) Indicate the study's design with a commonly used term in the title or the abstract	1	Line 1-2
		(*b*) Provide in the abstract an informative and balanced summary of what was done and what was found	1	Line 41-62
**Introduction**				
Background/rationale	2	Explain the scientific background and rationale for the investigation being reported	3-4	Line 73-135
Objectives	3	State specific objectives, including any prespecified hypotheses	5	Line 136-141
**Methods**				
Study design	4	Present key elements of study design early in the paper	6	Line 151-156
Setting	5	Describe the setting, locations, and relevant dates, including periods of recruitment, exposure, follow-up, and data collection	6-7	Line 148-181
Participants	6	(*a*) *Cohort study*—Give the eligibility criteria, and the sources and methods of selection of participants. Describe methods of follow-up<br>*Case-control study*—Give the eligibility criteria, and the sources and methods of case ascertainment and control selection. Give the rationale for the choice of cases and controls<br>*Cross-sectional study*—Give the eligibility criteria, and the sources and methods of selection of participants	7	Line 177-183
		(*b*) *Cohort study*—For matched studies, give matching criteria and number of exposed and unexposed<br>*Case-control study*—For matched studies, give matching criteria and the number of controls per case		
Variables	7	Clearly define all outcomes, exposures, predictors, potential confounders, and effect modifiers. Give diagnostic criteria, if applicable	9	Line 212-223
Data sources/ measurement	8*	For each variable of interest, give sources of data and details of methods of assessment (measurement). Describe comparability of assessment methods if there is more than one group	9-10	Line 218-247
Bias	9	Describe any efforts to address potential sources of bias	6	Line 144-150
Study size	10	Explain how the study size was arrived at	11	Line 260-262

Continued on next page

Quantitative variables	11	Explain how quantitative variables were handled in the analyses. If applicable, describe which groupings were chosen and why	10-11	Line 249-264
Statistical methods	12	(*a*) Describe all statistical methods, including those used to control for confounding	10-11	Line 248-264
		(*b*) Describe any methods used to examine subgroups and interactions	10-11	Line 254-258
		(*c*) Explain how missing data were addressed	12	Figure 2
		(*d*) *Cohort study*—If applicable, explain how loss to follow-up was addressed<br>*Case-control study*—If applicable, explain how matching of cases and controls was addressed<br>*Cross-sectional study*—If applicable, describe analytical methods taking account of sampling strategy		
		(*e*) Describe any sensitivity analyses		
**Results**				
Participants	13*	(a) Report numbers of individuals at each stage of study—eg numbers potentially eligible, examined for eligibility, confirmed eligible, included in the study, completing follow-up, and analysed	12	Figure 2
		(b) Give reasons for non-participation at each stage	12	Figure 2
		(c) Consider use of a flow diagram	12	Figure 2
Descriptive data	14*	(a) Give characteristics of study participants (eg demographic, clinical, social) and information on exposures and potential confounders	13	Line 303-314
		(b) Indicate number of participants with missing data for each variable of interest	12	Figure 2
		(c) *Cohort study*—Summarise follow-up time (eg, average and total amount)	12	Figure 2
Outcome data	15*	*Cohort study*—Report numbers of outcome events or summary measures over time	12	Figure 2
		Case-control study—Report numbers in each exposure category, or summary measures of exposure		
		Cross-sectional study—Report numbers of outcome events or summary measures		
Main results	16	(*a*) Give unadjusted estimates and, if applicable, confounder-adjusted estimates and their precision (eg, 95% confidence interval). Make clear which confounders were adjusted for and why they were included	14-15	Line 335-347
		(*b*) Report category boundaries when continuous variables were categorized	14-15	Line 335-347
		(*c*) If relevant, consider translating estimates of relative risk into absolute risk for a meaningful time period		

Continued on next page

Other analyses	17	Report other analyses done—eg analyses of subgroups and interactions, and sensitivity analyses	14-15	Line 335-347
**Discussion**				
Key results	18	Summarise key results with reference to study objectives	17	Line 352-372
Limitations	19	Discuss limitations of the study, taking into account sources of potential bias or imprecision. Discuss both direction and magnitude of any potential bias	24-26	Line 464-521
Interpretation	20	Give a cautious overall interpretation of results considering objectives, limitations, multiplicity of analyses, results from similar studies, and other relevant evidence	23-24	Line 440-462
Generalisability	21	Discuss the generalisability (external validity) of the study results	26	Line 510-518
**Other information**				
Funding	22	Give the source of funding and the role of the funders for the present study and, if applicable, for the original study on which the present article is based	27	Line 536-537

*Give information separately for cases and controls in case-control studies and, if applicable, for exposed and unexposed groups in cohort and cross-sectional studies.

Note: An Explanation and Elaboration article discusses each checklist item and gives methodological background and published examples of transparent reporting. The STROBE checklist is best used in conjunction with this article (freely available on the Web sites of PLoS Medicine at http://www.plosmedicine.org/, Annals of Internal Medicine at http://www.annals.org/, and Epidemiology at http://www.epidem.com/). Information on the STROBE Initiative is available at www.strobe-statement.org.
